# Clinical spectrum, diagnosis, cognitive outcomes and therapeutic strategies in pediatric Rasmussen syndrome: a 10-year cohort from a tertiary pediatric center

**DOI:** 10.1186/s13023-026-04357-8

**Published:** 2026-04-29

**Authors:** Verónica Cantarín-Extremera, Maria Ballarà-Petitbò, María Jiménez-Legido, Borja Esteso-Orduña, Inés Solís Muñiz, Silvia Cámara-Barrio, Ramón Cancho-Candela, Maria Angeles Pérez-Jiménez, Anna Duat-Rodriguez, Rosa Guerrero-López

**Affiliations:** 1 Pediatric Neurology Department, Hospital Infantil Universitario Niño Jesús, Avenida Menéndez Pelayo-65, Madrid, 28009 Spain; 2 Centre for Biomedical Network Research on Rare Diseases (CIBERER) (GCV 23/ER/03), Instituto de Salud Carlos III, Madrid, Spain; 3 C.S. Las Américas, Parla, Madrid, Spain; 4 Clinical Neuropsychology Unit, Psychiatrics and Clinical Psychology Department, Hospital Infantil Universitario Niño Jesús, Madrid, Spain; 5 Radiology Department, Infantil Universitario Niño Jesus Hospital, Madrid, Spain; 6 Psychiatrics Department, Hospital General Universitario Gregorio Marañón, Madrid, Spain; 7 Pediatric Neurology Department, Hospital Infantil Universitario Rio Hortega. Faculty of Medicine, Universidad de Valladolid, Valladolid, Spain; 8 Clinical Video-Electrophysiology Unit, Hospital Infantil Universitario Niño Jesús, Madrid, Spain; 9 Institute for Biomedical Research “Sols-Morreale”, CSIC-UAM, Madrid, Spain

**Keywords:** Rasmussen syndrome, Pediatric epilepsy, Cognitive decline, Immunotherapy, Hemispherectomy, Prognostic factors

## Abstract

**Background:**

Rasmussen syndrome (RS) is a rare, immune-mediated, progressive epileptic encephalopathy. A comprehensive understanding of its heterogeneous clinical course, along with the identification of prognostic and early determinants, is crucial for timely intervention. To this end, we retrospectively analyzed 21 pediatric RS patients (2014–2024). Collected data encompassed epidemiology, clinical course, cognition, neuroimaging, immunology, histopathology, and treatment. Primary outcomes included the time from onset to the appearance of different clinical symptoms and cerebral atrophy. Cognitive decline was assessed through longitudinal Full-Scale IQ (FSIQ) measurements.

**Results:**

Mean age at onset was 7.3 years; 52.4% were < 8 years. All patients presented with epilepsy as their initial manifestation and age at onset correlated with time to cognitive decline (*r* = 0.578, *p* = 0.049), motor symptoms (*r* = 0.568, *p* = 0.034) and *epilepsia partialis continua* (EPC) (*r* = 0.765, *p* = 0.004), identifying an age of 8 years as a cut-off to predict a different disease course. EPC occurred in 57.1% (median: 6.5 months) and its early onset was associated with a precocious appearance of motor deficit (*r* = 0.863, *p* < 0.001) and cerebral atrophy (*r* = 0.753, *p* = 0.007). The severity of cognitive decline was not related to age at disease onset, rapidity of development of cognitive impairment or baseline cognitive status. Four patients displayed atypical, slow-progressing forms with minimal deficits. Immunotherapy achieved partial or transient seizure control in most cases; rituximab was effective in 50%, particularly when started 2–5 years post-onset. Hemispherectomy yielded seizure freedom in 69.2%, with better postoperative FSIQ linked to higher preoperative FSIQ (*r* = 0.640, *p* = 0.025) and younger surgical age (*r* = −0.621, *p* = 0.003).

**Conclusions:**

Age at onset is a key prognostic factor in RS. The development of epilepsy and EPC, cognitive and motor impairment show a strong temporal association. The degree of cognitive decline does not parallel other clinical markers. Post-surgical cognitive outcomes are better in patients operated at a younger age and with greater cognitive preservation. We propose a clinical framework integrating immunotherapy selection and surgical planning to optimize outcomes and guide patient counseling.

## Background

Rasmussen Syndrome (RS), previously known as Rasmussen Encephalitis (RE) (ORPHA:1929), is a rare disease initially described in 1958 by Rasmussen and colleagues [1]. Currently, RS has been classified by the International League Against Epilepsy (ILAE) within epilepsy syndromes of variable age of onset, as an epileptic and/or developmental encephalopathy with progressive deterioration [2]. RS is a progressive, immune-mediated disorder in which T lymphocytes play a central role in the inflammatory response against the central nervous system (CNS), leading to the selective involvement of a single cerebral hemisphere [3]. This cell-mediated pathology is driven primarily by clonally expanded cytotoxic CD8+ T cells that destroy neurons and astrocytes through the release of granzyme B [4, 5]. The resulting inflammatory infiltrate also comprises complex populations of CD4+, γδ, and resident memory T cells (CD103+), which appear to perpetuate chronic unilateral neuroinflammation [6, 7]. In contrast, while various autoantibodies have been identified, they are currently regarded as an epiphenomenon resulting from chronic cell lysis rather than the primary pathogenic driver of the disease [8, 9].

This inflammation of one cerebral hemisphere leads to drug-resistant focal epilepsy, often complicated by recurrent status epilepticus and, in 50% of cases, associated to the onset of *epilepsia partialis continua* (EPC). Progressively, unilateral hemispheric atrophy develops resulting in progressive hemiplegia, hemianopsia, cognitive deterioration, sensory impairment, and, if the affected hemisphere is language-dominant, aphasia [10, 11]. The disease affects children with previously normal development, usually in the late toddler or school-age years with a median age of onset of 6 years, though cases with onset in adolescence or adulthood may account for up to 10%. There are no incidence or prevalence studies in Spain; however, two classical studies estimated the incidence at 2–4 cases per 10 million individuals aged 18 years or younger per year in Germany [12] and 1–7 cases per 10 million individuals aged 16 years or younger per year (a prevalence of 0.18 per 100,000 individuals) in the United Kingdom [13].

Diagnosis of this entity continues to rely on criteria established by Bien et al. [10], in the 2005 European consensus on the pathogenesis, diagnosis, and treatment of RS, which are structured into two parts: Part A, requiring the presence of focal seizures (with or without EPC), progressive cortical atrophy, and specific EEG or MRI findings; and Part B, which allows for diagnosis through histopathological confirmation or the presence of EPC associated with progressive atrophy, even in the absence of initial Part A clinical markers. These criteria were validated by Olson et al. in 2013 [14].

## Methods

The aim of this study is to analyze the epidemiological characteristics and clinical evolution of pediatric patients with RS, with particular emphasis on cognitive aspects and the factors that influence the disease course.

A retrospective and descriptive study was conducted on a consecutive sample of 21 patients diagnosed with RS between 2014 and 2024. All patients meeting the criteria established by Bien et al. [10] were included, regardless of whether they underwent surgery or were managed solely with medical treatment. The mean follow-up time from disease onset was 7 years and 5 months (range: 2 years and 8 months to 13 years and 2 months). Although 9 patients (42.8%) were transitioned to adult neurology services upon reaching 18 years of age, their data was analyzed up to that point and this did not preclude the assessment of the possible residual phase, because these patients were followed at our center with an average follow-up of 9 years and 11 months from disease onset.

Data analyzed included epidemiological variables (age at onset, diagnosis delay, sex, and a personal or family history of autoimmune diseases or epilepsy); general clinical features (symptoms prior to disease onset, such as infectious syndrome, headache, signs of recent pubertal development), seizure onset pattern, seizure types, development of EPC, motor disorders (movement abnormalities or hemiparesis), and cognitive decline; complementary tests (electroencephalography, brain magnetic resonance imaging –MRI-), microbiological, immunological, and genetic analyses (clinical exome sequencing was conducted, with variant analysis restricted to a curated panel of epilepsy-associated genes), treatment regimens employed (including surgery), stages of cortical histopathology according to Pardo et al. [15] (normal cortex -stage 0-, focal inflammation with mild neuronal loss -stage 1-, panlaminar inflammation and gliosis with increasing neuronal loss -stages 2–3-, end-stage cortical cavitation with minimal residual inflammation -stage 4-) and response to treatment based on clinical or radiological evolution. Additionally, response to surgery was evaluated using Engel classification [16]. A favorable response was defined as sustained improvement on the Engel Epilepsy Surgery Outcome Scale (i.e., Class I, II, or III) at follow-up within a six-month window after treatment initiation, allowing for one month of stabilization.

Given the neurodegenerative nature of the disease, the timing of symptom onset was considered an essential variable in analyzing the clinical progression of patients. Therefore, the time to the development of EPC, motor impairment, cognitive decline, and brain MRI-detected atrophy were defined as primary points of interest.

Cognitive impairment was assessed using the Full Scale Intelligence Quotient (FSIQ) from the WISC-V. Consistent with the aims of the study, FSIQ was used as an index of overall cognitive status and global cognitive decline over the disease course. FSIQ was selected as the primary cognitive outcome to ensure a uniform metric for within- and between-patient comparisons across timepoints. Both the initial (baseline) FSIQ and the pre-surgical FSIQ were analyzed to characterize cognitive functioning prior to intervention. A specific variable, “cognitive deterioration,” was defined as the difference between baseline and pre-surgical FSIQ, with the aim of estimating decline associated with disease progression rather than with surgical treatment.

Descriptive statistics were used to summarize the data. Categorical variables were presented as absolute frequencies and percentages, and continuous variables as mean and standard deviation (SD). The Shapiro-Wilk test was used to assess normality. For comparison of parametric continuous variables between two independent groups, the independent t-test was applied. When the assumptions of normality were not met, the Mann–Whitney U test was used to assess differences between groups. Associations between binary and continuous variables were examined using point-biserial correlation. Spearman’s rho was used to assess monotonic relationships between ordinal or non-normally distributed continuous variables. Pearson’s correlation was used for linear relationships between continuous variables. Differences in final cognitive outcomes (FSIQ scores) across groups defined by SR lateralization were analyzed using ANOVA. A linear regression analysis was conducted to explore predictors of postoperative functional deterioration (FSIQ score) including age at surgery, reporting the coefficient of determination (partial η^2^). Additionally, boxplots were used to visually represent clinical timelines in patients with RS. One set of analyses compared the age at onset or time to development of clinical events—such as epilepsy onset, motor deficits, cognitive decline, EPC, brain atrophy, hyperintensities on MRI, time until initiation of immunotherapy, and surgery—between patients diagnosed before and after 10 years of age (<120 months vs. ≥120 months). A second analysis compared the time from epilepsy onset to the first administration of rituximab between patients who showed clinical efficacy and those who did not. We used a 24-month threshold a priori as a clinically meaningful boundary between early and late rituximab initiation. A significance level of *p* < 0.05 was considered statistically significant. IBM SPSS version 25 and Microsoft Excel 2016 were used for the collection and analysis of data, and Python (v3.11) with the pandas, seaborn, matplotlib, and SciPy libraries for data visualization.

## Results

### Baseline characteristics

A total of 21 patients with RS were recruited between January 2014 and July 2024. Baseline characteristics are summarized in Table 1. It is important to note that there was no missing data across the cohort. All patients and their legal guardians were systematically interviewed regarding each of the clinical, personal, and family history characteristics listed. The frequencies and percentages reported in the table reflect only those patients who manifested the specific characteristic. Consequently, while some rows do not sum to the total *N* = 21, this indicates the absence of the trait in the remaining patients, rather than a loss of clinical records. Data completeness was 100% for all variables analyzed.

**Table 1:** Patient demographics and general characteristics (*N* = 21)

	N	(%)	Notes
Sex			
Women	15	71,40	
Men	6	28,60	
Affected hemisphere			
Left	10	47,60	
Right	11	52,40	
Family history			
Epilepsy	4	19,04	
Autoimmune disease	8	38,09	Thyroiditis (*N* = 1, mother), diabetes (*N* = 2, mother and father), vitiligo (*N* = 1, father and sister), psoriasis (*N* = 1, mother), coeliac disease (*N* = 1, father), uveitis (*N* = 1, grandmother), some autoimmune diseases in the same family (*N* = 1, lupus erythematosus –aunt-, myasthenia gravis -uncle-, thyroiditis -father and other uncle-).
Personal history			
Perinatal	3	14,28	Intrauterine growth restriction (*N* = 1), left middle cerebral artery infarction (*N* = 1), obstructive hydrocephalus caused by stenosis of the Sylvian aqueduct (*N* = 1)
Autoimmune disease	3	14,30	Ipsilateral uveitis (*N* = 1), Type I diabetes mellitus (*N* = 1), moderate allergic asthma (*N* = 1)
Previous/associated symptoms			
Preceding headache weeks/months before onset	4	19,00	
Precocious puberty	4	19,00	All girls. Onset 1–6 months before the onset of epilepsy
Infection previous/concurrent at onset	4	19,00	Fever of unknown origin (*N* = 2), upper respiratory tract infection (*N* = 1), gastroenteritis (*N* = 1)

Note: *N* = 21 for all variables. Frequencies represent only positive findings (presence of the feature); no data was missing

For the purposes of this study, disease onset was defined as the age at which the first seizure occurred. While we recognize that subclinical pathological processes and electroencephalographic changes likely precede this milestone (the ‘biological onset’), the first seizure represents the ‘clinical onset’ that systematically triggered medical evaluation and the initiation of the diagnostic workup in our cohort. In this regard, the mean age at symptom onset was 7 years and 3 months (range: 1 year and 9 months to 11 years and 11 months), with epilepsy being the initial clinical manifestation in all cases.

A notable diagnostic delay was observed, with a mean time of 21.62 months (range: 3 months to 4 years and 10 months) from seizure onset to fulfillment of the Bien et al. criteria [10] that enabled the diagnosis of RS. A subjective impression of shorter diagnostic delay has been noted in recent years; therefore, the mean time to diagnosis was compared between patients whose disease onset occurred in the last 5 years and those with earlier onset, yielding 17.44 months ±14.85 months versus 24.75 months ±17.23, respectively. However, this difference did not reach statistical significance (F = 0.957, *p* = 0.340). The patient with the longest diagnostic delay was the oldest case in the series and, despite presenting with a clinically overt disease, remained misdiagnosed with isolated CNS vasculitis for 4 years and 10 months.

### Clinical presentation

All patients initially presented with epileptic seizures, one of whom manifested as focal status epilepticus requiring admission to the Intensive Care Unit. Refractory epilepsy was confirmed in all patients except one, who experienced only three seizures during the first five months after epilepsy onset and subsequently developed progressive hemispheric atrophy and a biopsy compatible with stage 4 RS.

Twelve patients (57.1%) developed EPC at a mean time of 15.41 months (SD 17.27; median 6.5 months; range 1 month to 4 years) from epilepsy onset. The most affected region was the face (58%). In one patient, EPC extended to the vocal cords, severely impacting speech. In three patients, the functional impairment caused by EPC was managed with botulinum toxin injections in the affected anatomical areas (hemiabdomen and vocal cords in one case, right foot and hand in the other two, respectively). The age at EPC onset showed a significant correlation with the time to motor disorder onset (*r* = 0.863, *p* < 0.001) and the time to cognitive decline (*r* = 0.786, *p* = 0.048).

Regarding the onset pattern, five patients (23.8%) presented with what could be described as an “aggressive onset” or “rapidly progressive course”, characterized by refractory epilepsy within the first three months. Two of these patients were previously completely asymptomatic, while the other three reported frequent headaches beginning several weeks before seizure onset, and two of them also exhibited early pubertal development at presentation. Four patients developed EPC within the first six months after onset, while the fifth patient developed EPC after 25 months of disease progression. Correlation analysis revealed a significant negative correlation between “aggressive onset” or “rapidly progressive course” ‘cases and degree of cognitive decline (*r* = −0.588, *p* = 0.044), indicating that despite a more abrupt onset, these patients experienced less cognitive decline. However, no significant association was found between these cases and the age at onset, time from onset to hemiparesis or the time to surgery.

From a motor perspective, 13 cases (61.9%) developed progressive hemiparesis at a mean time of 18.15 months after disease onset, with severity correlating to epilepsy worsening. Among those who did not develop an established hemiparesis (38.1%), transient mild-to-moderate paresis was observed during seizure clusters, followed by recovery. One patient manifested this motor deficit as a movement disorder, presenting with hemidystonia one year after epilepsy onset.

Cognitive impairment and behavioral disturbances were other neurological manifestations assessed in all patients. Each patient underwent at least one standardized neuropsychological evaluation during disease progression, and 12 patients (57.14%) had successive assessments.

At the initial evaluation, 12 patients (57.14%) maintained normal cognitive levels, defined as an FSIQ score of 85 or above. This assessment occurred at a mean of 17.25 months after onset (median 17.5 months; range 3–40 months). Conversely, 9 patients (42.85%) already exhibited cognitive impairment at this first evaluation, conducted at a mean of 25 months post-onset (median 15 months; range 4–53 months). The lowest recorded FSIQ at this time was 59, corresponding to a disease duration of 51 months.

Over time, of the 12 patients who presented a normal FSIQ at the initial evaluation, 4 developed cognitive deterioration at a mean of 32.25 months after disease onset (median: 26.5 months; range: 20–56 months). Among the 8 patients in whom cognitive decline was not observed, 4 (mean FSIQ: 95) had no follow-up assessments available, and their initial evaluations were conducted at a mean of 17 months after onset (median: 18 months; range: 3–29 months). In the remaining 4 patients, FSIQ remained stable in subsequent assessments (mean FSIQ: 108), performed at a mean of 82.5 months after onset (median: 95 months; range: 29–111 months). Notably, of these last 4 patients, one had a relatively recent disease onset (29 months) and showed excellent seizure control, while the remaining 3 evolved as atypical RS cases.

The relationship between the degree of pre-surgical cognitive decline (considered significant when a drop of 15 or more points in FSIQ score was registered) and various clinical progression variables was explored. Only the initial FSIQ showed a significant correlation with cognitive decline (*r* = 0.585, *p* = 0.046). Importantly, no significant association was found with age at disease onset or time to onset of cognitive decline, meaning that an earlier onset of the disease or earlier appearance of cognitive impairment during the disease course did not correlate to a greater decline in FSIQ in the long term.

When exclusively analyzing the 13 patients who underwent surgical intervention, 7 of them (53.8%) exhibited significant cognitive deterioration in post-surgical assessment, while another—affected by right-hemisphere RS (non-dominant for language)—showed a slight improvement in FSIQ following surgery (from 78 to 87), maintaining overall cognitive functioning within the normal range, with only mild executive dysfunction.

A significant correlation was found between the post-surgical FSIQ and the pre-surgical longitudinal FSIQ (*r* = 0.640, *p* = 0.025), supporting an association between preoperative functional status and postoperative cognitive outcomes. Furthermore, a significant correlation was observed between an earlier age at the time of surgery and a better score in post-surgical FSIQ (*r* = −0.621, *p* = 0.003). No statistically significant associations were found between post-surgical FSIQ and other clinical variables.

In addition, 12 patients (57.1%) presented, at some point during their clinical course, with behavioral disturbances of varying severity and fluctuating course. One of these patients required admission to a psychiatric unit for specialized management of his symptoms.

It is noteworthy that age at onset correlated positively with time to motor symptoms (*r* = 0.568, *p* = 0.034) and time to cognitive decline (*r* = 0.578, *p* = 0.049), indicating that later presentation is associated with longer intervals before these deficits appear. Moreover, time to EPC onset correlated significantly with age at onset (*r* = 0.765, *p* = 0.004) and with time to the appearance of cerebral atrophy on neuroimaging (*r* = 0.753, *p* = 0.007).

**Fig. 1:**
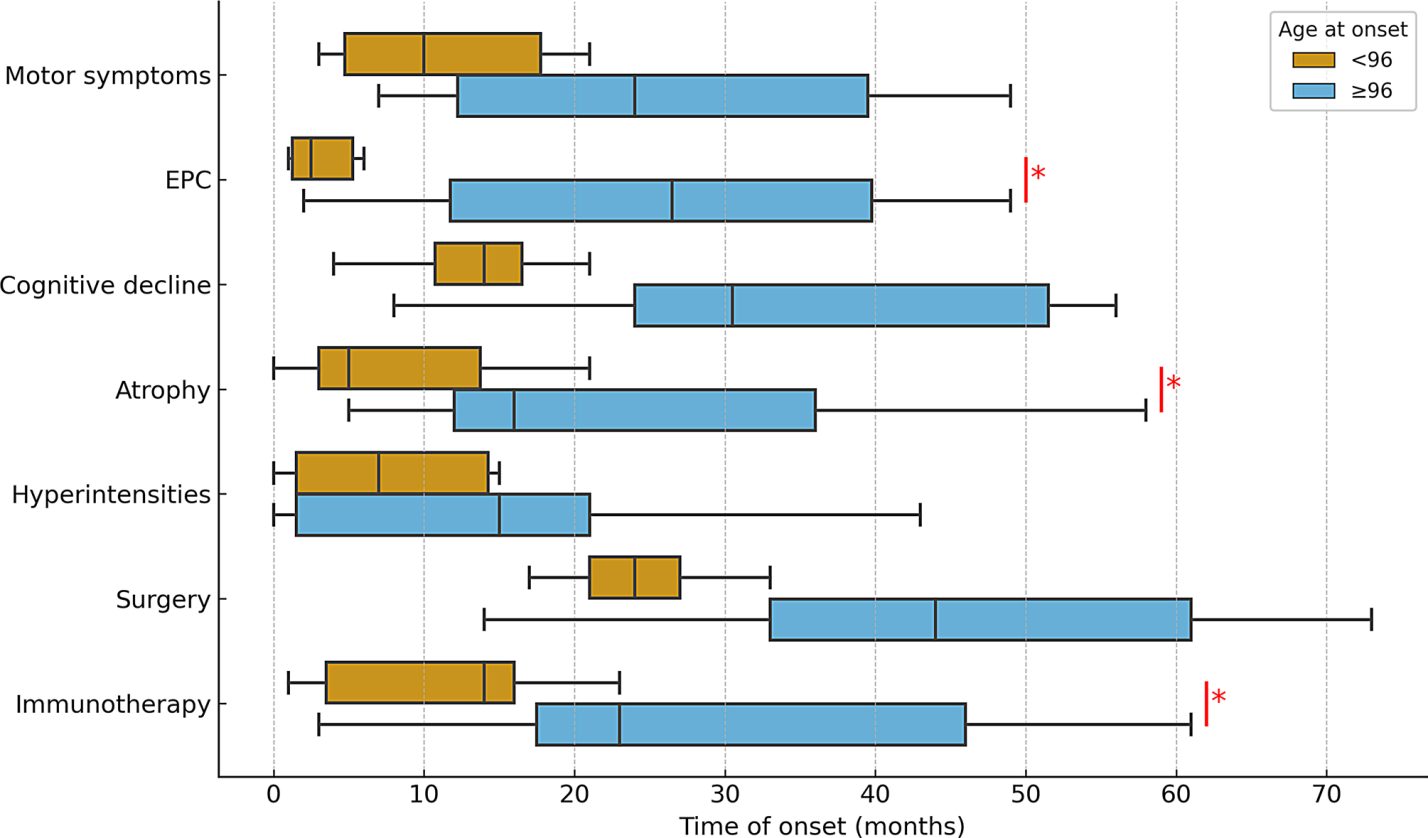
Clinical progression according to age at onset (<8 Years vs. ≥8 Years). Horizontal boxplots display the distribution of the time (in months) at which each variable appeared after disease onset in both groups depending on age at onset: <8 years (<96 months) or ≥8 years (≥96 months). Boxes represent the interquartile range (IQR), the line inside the box indicates the median, and whiskers extend up to 1.5 × IQR. Vertical red brackets with asterisks indicate statistically significant differences between groups (*p* < 0.05). Statistical comparisons were performed using the Mann–Whitney U test

When analyzing the disease evolution based on the age at onset of the disease, significant differences were observed in the clinical course between those who developed RS before age 8 years (*N* = 11) or after age 8 years (*N* = 10). This allowed stratification of the cohort according to age at onset (see Fig. 1).

Patients with disease onset before 8 years of age showed a significantly faster instauration of EPC and cerebral atrophy and an earlier initiation of immunotherapy compared with those with onset after 8 years, who exhibited a more delayed disease course. Although not statistically significant, earlier onset was also associated with a tendency towards earlier motor and cognitive impairment.

We highlight 4 patients who exhibited a clearly distinct clinical course, marked by fewer clinical manifestations and/or a more stable course of the disease. These have been classified as “atypical cases” and are detailed in Table 2.

**Table 2:** General characteristics of atypical cases of Rasmussen syndrome

Patient	Age at onset/Age at diagnosis	Affected hemisphere	Motor impairment onset	Cognitive impairment onset	EPC onset	Atrophy onset	Immunotherapy received	Follow-up time	Current treatment	Seizure control
P1	79/99	Right	95	-	1	13. Stabilization	Cs, IVIG, RTX	120	VPA, LCM, CNB	2–3/month, EPC
P2	96/132	Left	-	-	-	36. Stabilization	Cs, IVIG, Aza	120	BRV, VPA, CLB, Aza	2/month
P3	59/93	Left	-	-	-	0. Stabilization	Cs, IVIG, Aza	112	LEV	No seizures since 4 m after RS onset
P4	118/171	Right	129. Hemidystonia (MRI: right caudate nucleus atrophy)	53. Borderline IQ, non-progressive	-	53. Stabilization	Cs, IVIG, Aza	112	LCM, PER, ESL, CLB, Aza	1–2/week

All numbers express months. In the case of motor impairment onset, cognitive impairment onset, EPC onset and atrophy onset, the numbers express time in months from onset of the SR

Abbreviations: Cs: corticosteroids, IVIG: intravenous immunoglobulins, RTX: rituximab, Aza: azathioprine, VPA: valproate acid, LCM: lacosamide, CNB: cenobamate, BRV: brivaracetam, CLB: clobazam, LEV: levetiracetam, PER: perampanel, ESL: eslicarbazepine, IQ: intellectual quotient

The mean diagnostic delay in these cases was 3 years, compared to 1 year and 9 months in the overall cohort. Notably, the mean age at onset in these patients (7 years and 4 months) did not differ from that of the rest of the cohort. A moderate and significant positive correlation was found between atypical cases and the presurgical FSIQ (*r* = 0.598, *p* = 0.040), suggesting that atypical cases are associated with less degree of cognitive decline. No significant correlations were found between these cases and the presence of other variables.

### Diagnostic workup

The diagnostic evaluation of our cohort followed the hierarchical framework of the European Consensus [10]. Clinical findings, neuroimaging, and Video-EEG carried the greatest relative weight, as they constitute the core pillars for a definitive non-invasive diagnosis (Part A criteria). Video-EEG was essential not only for documenting seizure semiology but also for identifying unihemispheric slowing or unilateral seizure onset. Histopathological evaluation provided definitive confirmation, obtained either through diagnostic brain biopsy or, more frequently, through the study of surgical specimens (hemispherectomy). In contrast, laboratory investigations (including metabolic screening and CSF analysis) were not considered diagnostic markers for RS; their role was limited to excluding mandatory differential diagnoses.

#### Video-electroencephalography (video-EEG)

Video-EEG was performed in all patients both at disease onset and during follow-up. All showed the typical features described in the Bien et al. criteria [10], with slowed background activity in the affected hemisphere compared to the contralateral side and ipsilateral focal epileptiform abnormalities. During follow-up, two patients exhibited near normalization of electrical activity.

#### Neuroimaging

Cranial MRI was the primary imaging modality and was performed in all patients. The first MRI was performed at a mean time of 2.8 months after onset (median 2 months, range 0–14 months). In 8 cases (38%) the MRI was normal, 7 (33.3%) showed white matter hyperintensities on T2-FLAIR, 5 (23.8%) showed signs of hemispheric atrophy, and one (0.05%) exhibited both findings (hyperintensities and localized atrophy).

During disease progression, all patients developed hemispheric atrophy in a median time of 16,42 months after epilepsy onset (median 10,5 months, range 0–58 months). In contrast, only 15 cases (71,42%) showed white matter hyperintensities, in a median time of 10,2 months (median 5,5 months, range 0–43 months). Both cerebral atrophy (*U* = 0.0, *p* = 0.000) and T2 hyperintensities (*U* = 3.0, *p* = 0.001) showed statistically significant differences using the Mann-Whitney U test, with a shorter time from onset to the development of atrophy and hyperintensities in patients with disease diagnosis before 10 years of age.

A significant and positive correlation was observed between the time to appearance of cerebral atrophy and the time to onset of hemiparesis (*r* = 0.650, *p* = 0.016).

#### Laboratory tests

In cerebral spine fluid (CSF), analysis (17/21, 81%) revealed hyperproteinorrachia in one patient and positive oligoclonal bands in 5/13 (23.8%). Lymphoid population analysis (6/21) showed elevated CD4+ in 4 cases and elevated CD8+ in one, though variable sampling times (2 months–5 years post-onset) limit interpretation. Antineuronal antibodies (13/21) and interleukin levels (IL-1, IL-6, and TNF-α) (4/21) were unremarkable. In serum, autoimmunity screening (including ANA, C3, C4, rheumatoid factor, antithyroid antibodies and ASLO) (16/21, 76.2%) was abnormal in 8 (38.1%). Microbiological tests in CSF or serum were negative in all patients. Genetic testing (10/21) identified only variants of uncertain significance in *EFHC1, CACNA1A, SCN1A, KCNT1, PITRM1,* and *GUF1*.

#### Histological study

Histological analysis was performed in 14 patients (13 post-surgical specimens and one diagnostic biopsy). Findings compatible with RS were found in 12 cases: stage 1 in 5, stage 2 in 3, stage 3 in 2, and stage 4 in 2 patients. In one patient, the surgical specimen showed healthy tissue (considered stage 0), and in another patient, the stage was unknown due to surgery having been performed abroad. The tissue samples were obtained at variable disease stages, with a median of 25 months from disease onset (range 6 months to 8 years and 2 months). Notably, the two patients with the highest histological stage (stage 4) had markedly different clinical courses: one had a mild course with only 3 isolated seizures and no documented motor or cognitive decline at 33 months of follow-up (at the time of biopsy) (considered an atypical case, refer to Table 2), whereas the other patient presented with daily focal motor seizures, cognitive decline, and 21 months of disease evolution.

Epidemiological characteristics, treatment details, histological and clinical outcomes of these patients are summarized in Table 3.

**Table 3:** Disease characteristics of patients with available histological tissue samples

Patient	Age at onset (months)	Treatment details	Duration of illness prior to surgery/biopsy (months)	Hx stage	Outcome	
		Immunomodulation other than IVIG, IVMP			Seizures	Cognitive level (FSIQ)
P3	59	AZA	93	4	No	112
P5	20	RTX	24	1	Yes	88
P6	78	RTX, MM, PEX	6	1	Yes	60
P7	85	RTX, PEX, TCR	22	2	Yes	70
P8	119	TCR	13	1	Yes	61
P9	59	AZA	93	4	No	112
P10	40	AZA	27	1	Yes	70
P11	107	RTX	73	0	No	69
P12	76	-	17	3	No	57
P13	83	PEX	21	4	Yes	63
P14	120	RTX, IA	33	2	Yes	40
P15	104	MTX, ETANE	61	UNK	No	99
P16	57	-	98	2	Yes	66
P17	120	RTX	47	1	No	67
P18	64	RTX, ADA	25	3	No	50

Abbreviations: Adalimumab (ADA), Azathioprine (AZA), Etanercept (ETANE), Full-scale Intellectual Quotient (FSIQ), Histological (HX), Immunoadsorption (IA), Intravenous immunoglobulin (IVIG), Intravenous Methylprednisolone (IVMP), Methotrexate (MTX), Mycophenolate Mofetil (MM), plasmapheresis (PEX), Rituximab (RTX), Tacrolimus (TCR), Unknown (UNK)

### Treatment

Seizures were refractory to all antiseizure medications (ASMs) regimens used, except in one case, who experienced only three seizures controlled with levetiracetam. The mean number of ASMs administered per patient was 6.8. Additionally, 6 patients required continuous midazolam infusion due to refractory status epilepticus on one or more occasions. Overall, levetiracetam and valproic acid were the most frequently used ASMs, followed by clobazam and sodium channel inhibitors such as lacosamide and carbamazepine. Four patients received cenobamate as ASM, only two cases had a lightly better seizure control. None of the ASMs prevented disease progression in terms of radiological findings or clinical neurological deficits.

Immunomodulatory and immunosuppressive therapies were initiated at the time of diagnostic suspicion in all cases, including one patient in whom CNS vasculitis was initially suspected. First-line treatments for autoimmune encephalitis were used initially, mainly corticosteroids (primarily intravenous methylprednisolone and/or oral prednisone) and intravenous immunoglobulins. Improvement, mainly in seizure control, was observed in 17 patients (81%), which was temporary in 14 and sustained in 3 patients. In these latter cases, due to the observed efficacy, therapy was switched to azathioprine, maintaining the beneficial therapeutic effect. It is noteworthy that in a fourth patient, azathioprine did not lead to clear improvement, but its withdrawal was associated with the appearance of EPC within the following month. Plasmapheresis and/or immunoadsorption were employed in 4 patients (19%) during episodes of seizure worsening, showing efficacy in 2 cases (50%), although the effect was transient.

Second- and third-line immunomodulatory drugs were administered in 17 patients (81%), including rituximab (10 patients, 58.8%), mycophenolate mofetil/azathioprine (8 patients, 47%), tacrolimus (2 patients, 11.7%), anakinra (1 patient, 5.8%), etanercept (1 patient, 5.8%), and methotrexate (1 patient, 5.8%). In 2 cases (11.7%), more experimental therapies such as mesenchymal stem cell infusions were employed, showing marked improvement in seizure reduction; however, seizures recurred upon treatment discontinuation.

**Fig. 2:**
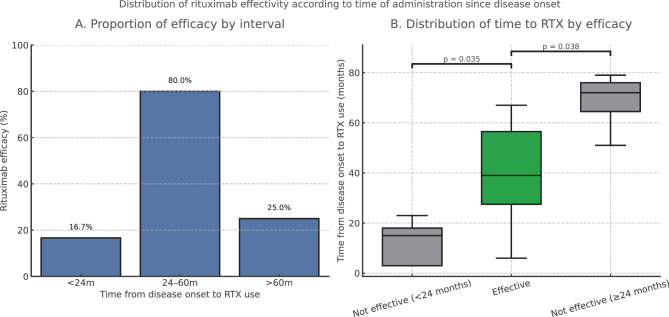
Distribution of rituximab (RTX) effectivity according to time of administration since disease onset. (**A**) Proportion of patients with effective response to RTX according to the interval between disease onset and RTX initiation (<24 months, 24–60 months, >60 months). (**B**) Distribution of the time from disease onset to RTX initiation stratified by treatment effect (effective in green; not effective in grey, subdivided into <24 and ≥24 months). Boxplots show median, interquartile range, and full range (whiskers). *p*-values correspond to Mann–Whitney U tests comparing each “not effective” subgroup with the “effective” group

Rituximab demonstrated notable efficacy in 50% of the patients treated, resulting in significant seizure improvement: 2 patients remained seizure-free for a period of 4–6 months, while the remainder experienced at least a 50% reduction in seizure frequency. This efficacy led to repeated rituximab administrations in 4 cases (twice in each) and one case received it three times; however, subsequent doses elicited a less favorable response compared to the initial administration. Analysis of the timing of rituximab efficacy indicated that the optimal response window occurred approximately between 2 and 5 years after epilepsy onset, with diminished responsiveness observed both before and after this period (see Fig. 2).

The time elapsed from disease onset to initiation of immunotherapy (IT) did not show a significant association with the time to onset of hemiparesis, cognitive deterioration, EPC or cerebral atrophy.

Surgical treatment was performed in 13 cases (62%), at a mean age of 10 years and 8 months, with a mean disease duration of 3 years and 9 months. The average delay from the diagnosis of RS to surgery was 2 years and 2 months. Complete seizure control (Engel IA) was achieved after the first surgery in 5 patients (38.5%). Of the remaining 8 patients, 6 required reoperation (46.1%), resulting in an additional 3 patients becoming seizure-free. Overall, 9 patients (69.2%) remained seizure-free at the end of the study (Engel IA), while the other 4 experienced occasional episodes primarily related to infections (Engel IIA).

Statistical analyses revealed that patients with left hemisphere involvement tended to undergo surgery sooner after clinical onset (rb = 0.595; *p* = 0.032) compared to those with right hemisphere involvement. The median time to surgery was 13.5 months in the left hemisphere group versus 20 months in the right hemisphere group.

Moreover, surgical outcomes were clearly more favorable when the procedure was performed at an earlier age (F = 97.867; *p* = 0.000), accounting for 85.2% of the variance in the final/postoperative FSIQ (partial η^2^ = 0.502).

## Discussion

We present the first Spanish cohort of patients with RS followed at a third-level hospital dedicated exclusively to pediatric care.

### Epidemiological characteristics and external validity

We acknowledge that our cohort size (*N* = 21) represents a limitation for the statistical power of subgroup analyses. However, given the extreme rarity of RS, our cohort is representative and comparable to other international series from reference centers in Toronto [17] as do centers in India [18]. The mean age at onset in our series was approximately 7 years, with 9.5% of patients older than 10 years, which is consistent with previously published data [19–21] as British and German cohorts [3, 22]. Likewise, the frequency of EPC in our sample (57.1%) falls within the range reported in international literature, which oscillates between 50 and 92% depending on the series [3, 23]. Regarding surgical outcomes, our seizure freedom rate (69.2%) is consistent with the 63–80% success rate reported in major meta-analyses and series from high-complexity centers in North America and Europe [17, 24]. This concordance reinforces the external validity of our data, suggesting that the natural history and therapeutic response in the Spanish population do not differ significantly from other European settings.

#### Age at onset as a prognostic and stratifying factor

There is no single, strict age cutoff to distinguish between pediatric-onset, adolescent-onset, or adult-onset RS, which appear to have clinically relevant distinctions [25]. In our cohort, younger age at onset was associated with an earlier development of EPC and motor deficits, as well as with an earlier onset of cognitive impairment (though not with greater severity of cognitive decline). This observation is further supported by the differences observed in the clinical profiles of our patients when applying an 8-year age cutoff at disease onset suggesting the existence of an early-onset phenotype characterized by a more aggressive and rapidly progressive disease course, with structural involvement, complex neurological symptoms, and therapeutic escalation at significantly younger ages. Otherwise, disease onset after the age of 8-year may be associated with a slower progression and a longer period free from severe neurological deficits. These findings support the hypothesis that age at clinical disease onset has prognostic and organizing value for disease evolution, possibly reflecting different pathophysiological mechanisms, and advocate for the systematic inclusion of age at onset as a key stratifying variable in the phenotypic classification of RS patients. Recognizing this distinction may aid in early prognostication, optimize the timing and intensity of therapeutic interventions, and improve patient and family counseling.

However, it is important to note that, in our study, RS presented in all cases with epilepsy as the initial symptom. Consequently, the variable “age at disease onset” effectively corresponds to “age at epilepsy onset.” This distinction is relevant, as it remains unclear whether the observed associations reflect the true onset of the disease or are specifically related to epilepsy. Addressing this issue would require studies including patients whose initial clinical manifestations are non-epileptic.

### Immunogenetic background and shared autoimmune predisposition

Considering the immune/inflammatory basis of RS, we highlight the presence of other immune-mediated diseases of varying nature in 3 patients from our cohort (14.3%): diabetes mellitus, uveitis, and moderate allergic asthma. This finding is in line with that reported by Amrom et al., who described a series of 29 patients with a 13.8% prevalence of comorbid autoimmune disease [26]. The diagnosis of uveitis (either ipsilateral or bilateral) prior to or concurrent with the onset of epilepsy is particularly relevant for raising suspicion of RS [27–31]. Similarly, 8 patients (38%) had a family history of immune-mediated disorders, 7 of whom had first-degree relatives affected. This association with familial immune pathology has been previously reported and suggests the involvement of common genetic factors, such as HLA haplotypes, in both conditions [32, 33]. Detailed medical and family histories of autoimmune diseases, along with molecular analysis, would be required to confirm the evidence for a shared immunogenetic predisposition between RS and common immune-mediated disorders.

### Clinical phenotypes and disease progression

#### Atypical cases

The natural history of RS is classically divided into three stages: prodromal, active, and residual [22, 34]. However, our findings underscore the clinical difficulty in defining clear boundaries between these phases. While the prodromal stage is often described as paucisymptomatic, 19% of our patients experienced intense headaches and another 19% presented with precocious puberty months before the first seizure. We argue that these symptoms, although sporadically reported [35, 36], may represent other type of ‘red flags’ of early subclinical neuroinflammation. This is supported by recent evidence showing that resident memory T cells (CD103+) and microglial nodules are present in brain tissue even at the earliest clinical stages [6, 7]. Furthermore, 23.8% of our cohort exhibited an ‘aggressive onset’ or a ‘rapidly progressive course’, effectively bypassing a recognizable prodromal phase and entering directly into a robust active inflammatory stage. This phenotypic variability—from insidious to aggressive presentations—likely reflects the intensity of the underlying cytotoxic CD8+ T-cell response and highlights the need for a high index of suspicion before permanent neurological deficit or refractory epilepsy is established.

While the 2005 international criteria do not formally define prognostic subtypes, medical literature uses the term ‘Atypical RS’ to describe cases that diverge from the classic clinical course with fewer classic symptoms or unusual presentation, or an absence or very late onset of seizures, or presentations dominated by movement disorders—such as dystonia or chorea—rather than epilepsy [37–39]. This feature is frequently observed in adult-onset RS but also present in this pediatric subpopulation [40, 41]. The four patients identified in our cohort as ‘atypical’ align with descriptions of ‘benign’ or ‘non-progressive’ variants proposed by Rizek et al. (2014) [42], who suggest that the absence of EPC is a key indicator of a more stable or slowly progressive course [36, 42]. Reports by Gorman et al. (2015) [39] further emphasize that seizures are not an essential feature for diagnosis if progressive atrophy and active inflammation are documented, describing a milder clinical course in this subgroup. In our sample, this phenotype was not associated with an older age at onset, suggesting that other factors likely modulate this variability in disease progression. As was foreseeable, we did observe a greater diagnostic delay in these patients, as the absence of all the expected clinical features of the disease complicates the diagnosis. Finally, we were unable to identify a correlation with other clinical variables that could predict which patients would follow this course of progression. This last point should be a priority in current research, as recognizing early markers—radiological, clinical, and analytical (such as interleukins and immune cell populations)—that forecast disease progression would allow for a much more individualized treatment approach and facilitate decision-making. Nonetheless, this topic lies beyond the scope of the present study [9, 20, 34, 43, 44].

Among these atypical cases, one patient is noteworthy for developing an hemicorporal choreo-dystonic movement disorder one year after epilepsy onset, without associated EPC. This presentation has been previously described in both pediatric and adult patients, with hemidystonia or hemiathetosis even constituting the first symptom of the disease [45, 46]. When present, this manifestation should prompt consideration of RS, especially in the context of other compatible findings.

#### Epilepsy

Focal epilepsy, often drug-resistant, is a cardinal feature of RS. Within this spectrum, EPC, as a specific form of epilepsy, represents a distinctive manifestation of this disorder. In our series, EPC was observed in 57.1% of patients, developing within a period of 15 months from epilepsy onset. This aligns with previously reported data, which indicate a prevalence of 37–92% in patients with RS [10, 19], with a mean time to onset of 31 months (12.5 months in pediatric cases versus 80 months in adult-onset RS, reflecting the typically more aggressive course of the disease in pediatric-onset cases). Furthermore, in our study, earlier onset of EPC was associated with earlier development of motor and cognitive decline as well as radiological atrophy. This suggests that the sequence of symptom emergence in RS is highly interconnected and that the appearance of EPC may either trigger or serve as a marker of a more rapidly progressive phase of the disease.

#### Motor function

Regarding motor function, hemiparesis typically develops within 12 to 36 months from disease onset [18, 23, 47] which is consistent with the 18-month median observed in our study. Initially, motor deficits may be subtle, manifesting only in the postictal phase or fluctuating with seizure burden, as observed in our cohort. Over time, weakness becomes progressive, permanent, and more pronounced. In our study, a relationship was found between the time to hemiparesis onset and the appearance of cerebral atrophy, suggesting a parallel trajectory between motor deterioration and structural brain damage as previously described by other authors [23, 38].

#### Cognitive evaluation

Cognitive decline in RS primarily manifests as deficits in memory, attention, and executive functions [11], typically emerging between 4 and 36 months after the onset of initial RS symptoms (mean 11.1 ± 9.4 months) [47], comparable to what was registered in our cohort (25 months). Literature indicates that the progression of cognitive decline appears to correlate with epilepsy severity [19, 48, 49]. In our study, the onset of cognitive decline was temporally associated with development of EPC—similarly to motor dysfunction—potentially explaining their parallel emergence during periods of heightened disease activity. Conversely, the severity of cognitive decline did not correlate with disease aggressiveness, as measured by the rate of symptom onset, age at disease onset, time to initiation of immunotherapy, or the presence of cerebral atrophy on MRI.

It is noteworthy that the degree of cognitive impairment was not even correlated with the time of onset of this cognitive decline, contrary to what might be expected, implying that earlier cognitive involvement does not necessarily lead to a greater decrease in FSIQ. And, also, higher baseline cognitive reserve did not protect from deterioration, indicating that all RS patients, if left untreated, may reach a comparable level of cognitive impairment. This indicates that currently unknown factors, beyond the mere temporal course of the disease or the initial cognitive reserve, act as modulators of the intellectual decline and highlights the importance of close neuropsychological follow-up in these patients, given that cognitive evolution does not correlate with other clinical or radiological parameters that could serve as indicators. Several mechanisms may account for this dissociation. First, heterogeneous immunopathological processes could differentially affect cortical networks, producing variable cognitive outcomes independent of disease duration. Second, the vulnerability of specific neural circuits, rather than the chronological onset of decline, may determine the severity of impairment. Moreover, while higher cognitive reserve may initially buffer symptoms, progressive neuroinflammation and epileptic activity may ultimately overcome compensatory mechanisms, leading to a convergence toward comparable levels of dysfunction. Taken together, these considerations highlight the multifactorial nature of cognitive deterioration in RS and reinforce the need for close neuropsychological monitoring, as conventional clinical or radiological parameters fail to capture the complexity of its progression.

It is well established that the only treatment shown to halt disease progression in RS is hemispherectomy. Despite its significant sequelae, this procedure can lead to overall neuropsychological improvement [50]. Our study demonstrates that postoperative FSIQ depends on preoperative FSIQ—that is, better cognitive status prior to surgery predicts better postoperative outcomes—and that younger age at the time of surgery correlates with higher postoperative FSIQ, likely reflecting greater cerebral plasticity. Early intervention—characterized by shorter epilepsy duration and younger age at surgery—has been associated with better cognitive outcomes [19, 34, 51]. These findings are critical for the challenging decision-making process in the therapeutic management of RS patients.

### Therapeutic strategies

Finally, one of the most medically relevant issues—and one in which many gaps still remain—is the treatment regimen for these patients. Follow-up of seizures, hemiparesis, cognitive performance and atrophy on neuroimaging can all be used to monitor clinical progress and treatment efficacy in these patients.

#### Antiseizure medication

ASMs have limited efficacy in RS and there is no ASM showing superiority in the management of seizures. In our series only one patient took a single ASM (levetiracetam), all other patients suffered drug-resistant epilepsy. Recent reports have highlighted the potential of new-generation ASMs, such as Cenobamate, in achieving significant seizure reduction in refractory RS cases [52]. However, it is important to distinguish between symptomatic seizure control and disease modification. In our series and based on current immunological understanding, ASMs—including potent sodium channel blockers—do not appear to halt the progressive cortical atrophy or the accumulation of neurological deficits. Consequently, while these drugs are invaluable for managing seizure burden, they should not be considered a substitute for immune-modulating or surgical interventions aimed at curbing the primary pathogenic process.

#### Immune treatment

Treatments targeting the immune system are recommended especially in the early stages of the disease, since in the residual phase structural damage likely predominates as the main cause of clinical manifestations rather than the inflammatory cascade. Immunotherapy can play a particularly important role in patients with slow disease progression, mild deficits, and not eligible for surgery or when appropriateness of surgery is less evident [34].

In line with previous publications [20, 53] and based on our own experience with this 10-year cohort, we offer a suggested clinical framework for management. This scheme is intended to provide a practical reference, particularly for patients with slow disease progression or where surgical timing is less evident, acknowledging that individualization remains paramount given the heterogeneity of RS. We consider that first-line treatment should include corticosteroids, with or without adjunctive immunoglobulins, as they act on both the innate and adaptive immune systems. These agents have been shown to reduce the expression of pro-inflammatory interleukins and to inhibit multiple co-stimulatory pathways. If clinical improvement is observed after 3–6 months of this treatment, the introduction of azathioprine or mycophenolate mofetil should be considered, along with the gradual withdrawal of the previous agents over a period of 6–9 months. This regimen has proven effectiveness in 3 of our patients. Conversely, if no clinical improvement is observed within the first 3–6 months, treatment targeting cytokine blockade—specifically those known to be involved in the pathophysiology of RS—should be considered, such as anakinra (IL-1 receptor antagonist) [54, 55], tocilizumab (IL-6 receptor antagonist) [56] or adalimumab/infliximab (TNF-α inhibitor) [57, 58]. At present, there is insufficient evidence to support the superiority of one agent over another, or to guide therapeutic decisions based on cytokine levels. In our series, anakinra was used in only one patient with highly refractory epilepsy (and normal IL-6 levels in CSF), without clear evidence of efficacy.

Rituximab has also been tested in RS. It has anti-CD20/B-cell effect, but it is noteworthy that B-cells also have a role in T-cell activation through their antigen-presenting and cytokine-producing activities. In our series, the effectiveness rate was 50%, primarily evidenced by an improvement in seizure control [59]. Notably, the best response to rituximab was observed when administered between 2 and 5 years after disease onset. This finding is particularly relevant considering the growing emphasis on precision medicine, which aims to identify the cytokines or cell populations most activated at each stage of the disease to enable targeted immunotherapy. This observation may indicate that during this period, B lymphocytes assume a primary inflammatory role (either per se or through interaction with other immune mechanisms), potentially helping to establish a temporal framework for the various immune system components reported as important in the pathophysiology of RS [53]. Nonetheless, this finding requires confirmation through more focused studies, as drawing definitive conclusions is challenging—especially given that the response to rituximab is likely influenced by multiple factors, which may also differ between pediatric and adult patients [53, 60].

Plasmapheresis and immunoadsorption should be used in times of worsening seizures and even in situations of status epilepticus [53].

A promising early-stage drug would be natalizumab, by blocking the inflammatory cytokines and T-cell migration through the blood brain barrier [53]. The study by Kebir et al. [61] described an experimental mouse model with induced RS in which prophylactic administration of α4-integrin blockers (such as natalizumab) before the clinical onset of the disease prevented the development of epilepsy. This effect was not observed when the drug was administered after the onset of seizures, despite a reduction in inflammatory cytokines and T lymphocytes in the CNS in both cases. This suggests that, once the disease is established, the reduction of T cell infiltration into the brain is not sufficient by itself to decrease seizure frequency, and the persistence of epilepsy in this phase likely depends on mechanisms beyond lymphocyte infiltration into the CNS.

Although the clinical response to immunotherapy in RS is well established, its role within the therapeutic algorithm remains under constant reevaluation. Given that its efficacy is often partial or temporary, it is essential that immunotherapy does not delay surgical intervention in patients who are clear candidates for surgery and could benefit from it. Therefore, understanding the time window during which immunotherapy may be effective in RE is crucial. In our study, a longer delay in initiating immunotherapy was not significantly associated with a faster development of motor impairment, cognitive decline, development of EPC, or the onset of cerebral atrophy. However, these results should be interpreted with caution. We believe two hypotheses could explain these findings: the small sample size and/or high heterogeneity—most likely the latter—or that the clinical impact of delayed immunotherapy initiation is neither linear nor cumulative, but instead depends on other modulating variables such as clinical, structural, or genetic factors.

#### Use of botulinum toxin

We would like to highlight the potential utility of botulinum toxin in the management of EPC associated with RS. In the last three patients in our series presenting with EPC, targeted administration of botulinum toxin to the affected areas led to the recovery of hand function and gait in two patients, respectively. Additionally, it provided effective pain relief occasioned by hemiabdominal EPC and improved speech disturbances due to vocal cord involvement in another case. The use of botulinum toxin in RE has previously been reported [62, 63]; however, we believe it should be more frequently considered as a therapeutic option in such cases.

#### Surgery

Despite efforts to improve immunological treatments, surgery remains the most effective therapeutic option, with recent publications reporting seizure freedom rates ranging from 48% to 83% at five-year follow-up [20]- a figure very similar to that observed in our series (69.2%). Additionally, we found that patients with left hemisphere involvement underwent surgery earlier. This finding may reflect a greater clinical urgency or a lower tolerance for symptom progression when the left hemisphere, which is often dominant for language, is affected.

### Phatological findings

In our study, no correlation was found between neuropathological findings and disease duration or severity. We hypothesize that this low correlation may be explained by the patchy nature of the pathology characteristic of this condition [15], or alternatively, that no true clinicopathological correlation exists. Other authors have reported associations between histological stage and age at onset, longer epilepsy duration, or hemiparesis [15, 64]. It could be speculated that patients who received more aggressive or earlier immunotherapy might exhibit less cerebral inflammation (reflected by a lower histological stage); however, this relationship was not observed in our study, consistent with findings reported in other publications [18].

## Limitations

This study has the inherent limitations of a retrospective design. Cognitive assessments, as well as laboratory or radiological evaluations, were performed at different time points for each patient, depending on their clinical manifestations and/or the indication of the attending pediatric neurologist. This variability may reduce the temporal accuracy of the findings. Moreover, advanced radiological techniques capable of quantifying atrophy and detecting subtle changes would be necessary.

## Conclusions

Through this study, we intend to carry out a detailed longitudinal analysis of pediatric patients with RS, offering a clearer understanding of phenotypic variability and its influencing factors.

It is essential to actively incorporate the evaluation of a personal or family history of autoimmune disorders and newly onset symptoms (such as headache or precocious puberty), as these may serve as early clinical markers of an underlying immunological basis. Expanding immunological characterization through the analysis of neuroinflammatory markers in serum and cerebrospinal fluid is a priority, both for diagnostic purposes and to further our understanding of the pathophysiological mechanisms of RE.

Clinical presentation is highly variable, with atypical cases that can make diagnosis challenging. Age at onset may modulate both clinical and radiological progression and should be considered as a stratifying variable. Moreover, epilepsy remains the central axis of disease, and its evolution is critically interconnected with the rapidity of motor and cognitive dysfunction. The severity of cognitive decline is not related to age at onset, rapidity of cognitive impairment onset or baseline cognitive status, indicating that other variables may play a role in determining cognitive prognosis. Post-surgical cognitive outcomes are better in patients who undergo surgery at a younger age and who have higher pre-surgical cognitive level.

Consequently, continuous clinical and neuropsychological monitoring is essential to guide therapeutic decisions, including surgical indications. Despite the lack of standardized therapeutic protocols, we propose a suggested clinical framework for immunotherapy and surgical planning based on our experience, which may serve as a useful reference in clinical practice.

## Data Availability

The data that support the findings of this study are not openly available due to reasons of sensitivity and are available from the corresponding author upon reasonable request.

## References

[1] Rasmussen T, Olszewski J, Lloyd-Smith D. Focal seizures due to chronic localized encephalitis. Neurology. 1958;8(6):435–435. 10.1212/WNL.8.6.435. 13566382 10.1212/wnl.8.6.435

[2] Riney K, Bogacz A, Somerville E, Hirsch E, Nabbout R, Scheffer IE, et al. International league against epilepsy classification and definition of epilepsy syndromes with onset at a variable age: position statement by the ILAE task force on nosology and definitions. Epilepsia. 2022, Jun;63(6):1443–74. 10.1111/epi.17240. PubMed PMID: 35503725. 35503725 10.1111/epi.17240

[3] Varadkar S, Bien CG, Kruse CA, Jensen FE, Bauer J, Pardo CA, et al. Rasmussen’s encephalitis: clinical features, pathobiology, and treatment advances. Lancet Neurol. 2014;13(2):195–205. 10.1016/S1474-4422(13)70260-6. PubMed PMID: 24457189. 24457189 10.1016/S1474-4422(13)70260-6 PMC4005780

[4] Bauer J, Bien CG, Lassmann H. Rasmussenʼs encephalitis: a role for autoimmune cytotoxic T lymphocytes. Curr Opin Neurol. 2002;15(2):197–200. 10.1097/00019052-200204000-00012. PubMed PMID: 11923635. 11923635 10.1097/00019052-200204000-00012

[5] Schwab N, Bien CG, Waschbisch A, Becker A, Vince GH, Dornmair K, et al. CD8+ T-cell clones dominate brain infiltrates in Rasmussen encephalitis and persist in the periphery. Brain. 2009;132(5):1236–46. 10.1093/brain/awp003. PubMed PMID: 19179379. 19179379 10.1093/brain/awp003

[6] Owens GC, Chang JW, Huynh MN, Chirwa T, Vinters HV, Mathern GW. Evidence for resident memory T cells in Rasmussen Encephalitis. Front Immunol. 2016;7(FEB). 10.3389/fimmu.2016.00064. PubMed PMID: 26941743. 10.3389/fimmu.2016.00064 PMC4763066 26941743

[7] Mair KM, Guggenberger V, Verdú de Juan L, Köck U, Lassmann H, Liblau RS, et al. The dynamics of brain T cell populations during the course of rasmussen encephalitis: from expansion to exhaustion. J Neuroinflammation. 2025;22(1). 10.1186/s12974-025-03477-5. PubMed PMID: 40506704. 10.1186/s12974-025-03477-5 PMC12164096 40506704

[8] Samanci B, Tekturk P, Tüzün E, Erdaǧ E, Kinay D, Yapici Z, et al. Neuronal autoantibodies in patients with Rasmussen’s encephalitis. Epileptic Disord. 2016;18(2):204–10. 10.1684/epd.2016.0829. PubMed PMID: 27248684. 27248684 10.1684/epd.2016.0829

[9] Tang C, Luan G, Li T. Rasmussen’s encephalitis: mechanisms update and potential therapy target. Ther Adv Chronic Dis. 2020;11. 10.1177/2040622320971413. 10.1177/2040622320971413 PMC7705182 33294146

[10] Bien CG, Granata T, Antozzi C, Cross JH, Dulac O, Kurthen M, et al. Pathogenesis, diagnosis and treatment of Rasmussen encephalitis: a European consensus statement. Brain. 2005;128(3):454–71. 10.1093/brain/awh415. PubMed PMID: 15689357. 15689357 10.1093/brain/awh415

[11] Granata T, Andermann F. Rasmussen encephalitis. Handb Clin Neurol. 2013;111:511–19. 10.1016/B978-0-444-52891-9.00054-3. PMID: 23622199. 23622199 10.1016/B978-0-444-52891-9.00054-3

[12] Bien CG, Tiemeier H, Sassen R, Kuczaty S, Urbach H, Von Lehe M, et al. Rasmussen encephalitis: incidence and course under randomized therapy with tacrolimus or intravenous immunoglobulins. Epilepsia. 2013;54(3):543–50. 10.1111/epi.12042. PMID: 23216622. 23216622 10.1111/epi.12042

[13] Lamb K, Scott W, Mensah A, Robinson R, Varadkar S, Cross J. Prevalence and clinical outcome of Rasmussen encephalitis in children from the United Kingdom. Dev Med Child Neurol. 2013;55(suppl 1):14.

[14] Olson HE, Lechpammer M, Prabhu SP, Ciarlini PDSC, Poduri A, Gooty VD, et al. Clinical application and evaluation of the Bien diagnostic criteria for Rasmussen encephalitis. Epilepsia. 2013;54(10):1753–60. 10.1111/epi.12334. PMID: 23980696. 23980696 10.1111/epi.12334

[15] Pardo A, Vining PG, Guo L, Skolasky RL, Carson S, Freeman M. The pathology of Rasmussen syndrome: stages of cortical involvement and neuropathological studies in 45 hemispherectomies. Epilepsia. 2004;45(5):516–26. 10.1111/j.0013-9580.2004.33103.x. 15101833 10.1111/j.0013-9580.2004.33103.x

[16] Engel J. Update on surgical treatment of the epilepsies. Summary of the Second international palm desert conference on the surgical treatment of the epilepsies (1992). Neurology. 1993;43(8):1612–1612. 10.1212/wnl.43.8.1612. PMID: 8102482. 8102482 10.1212/wnl.43.8.1612

[17] Hoffman CE, Ochi A, Snead OC, Widjaja E, Hawkins C, Tisdal M, et al. Rasmussen’s encephalitis: advances in management and patient outcomes. Childs Nerv Syst. 2016;32(4):629–40. 10.1007/s00381-015-2994-x. PMID: 26780781. 26780781 10.1007/s00381-015-2994-x

[18] Pradeep K, Sinha S, Mahadevan A, Saini J, Arivazhagan A, Bharath RD, et al. Clinical, electrophysiological, imaging, pathological and therapeutic observations among 18 patients with Rasmussen’s encephalitis. J Clin Neurosci. 2016;25:96–104. 10.1016/j.jocn.2015.05.062. PMID: 26675623. 26675623 10.1016/j.jocn.2015.05.062

[19] Cay-Martinez KC, Hickman RA, McKhann GM, Provenzano FA, Sands TT. Rasmussen Encephalitis: an update. Semin Neurol. 2020;40(02):201–10. 10.1055/s-0040-1708504. PMID: 32185790. 32185790 10.1055/s-0040-1708504

[20] Fornari Caprara AL, Rissardo JP, Nagele EP. Rasmussen Encephalitis: clinical features, pathophysiology, and management strategies—a comprehensive literature review. Medicina. 2024;60(11):1858. 10.3390/medicina60111858. PubMed PMID: 39597043. 39597043 10.3390/medicina60111858 PMC11596482

[21] Kumar A, Krishnani H, Pande A, Jaiswal S, Meshram RJ. Rasmussen’s encephalitis: a literary review. Cureus. 2023;15(10). 10.7759/cureus.47698. PMID: 38022088. 10.7759/cureus.47698 PMC10676233 38022088

[22] Bien CG, Widman G, Urbach H, Sassen R, Kuczaty S, Wiestler OD, et al. The natural history of Rasmussen’s encephalitis. Brain. 2002;125(8):1751–59. https://www.scioncorp. 12135966 10.1093/brain/awf176

[23] Caraballo RH, Fortini S, Cersósimo R, Monges S, Pasteris MC, Gomez M, et al. Rasmussen syndrome: an Argentinean experience in 32 patients. Seizure. 2013;22(5):360–67. 10.1016/j.seizure.2013.02.003. PMID: 23466213. 23466213 10.1016/j.seizure.2013.02.003

[24] Freeman JM. Rasmussen’s syndrome: progressive autoimmune multi-focal encephalopathy. Pediatr Neurol. 2005;32(5):295–99. 10.1016/j.pediatrneurol.2004.12.002. PMID: 15866428. 15866428 10.1016/j.pediatrneurol.2004.12.002

[25] Villeneuve N, Lépine A, Girard N, Guedj E, Daquin G. Rasmussen’s encephalitis: early diagnostic criteria in children. Rev Neurol. 2022;178(7):666–74. 10.1016/j.neurol.2022.03.012. PMID: 35568516. 35568516 10.1016/j.neurol.2022.03.012

[26] Amrom D, Kinay D, Hart Y, Berkovic SF, Laxer K, Andermann F, et al. Rasmussen encephalitis and comorbid autoimmune diseases: a window into disease mechanism? Neurology. 2014;83(12):1049–55. 10.1212/WNL.0000000000000791. PMID: 25142901. 25142901 10.1212/WNL.0000000000000791 PMC4166360

[27] Fauser S, Elger CE, Woermann F, Bien CG. Rasmussen encephalitis: predisposing factors and their potential role in unilaterality. Epilepsia. 2022;63(1):108–19. 10.1111/epi.17131. PMID: 34820830. 34820830 10.1111/epi.17131

[28] Fukuda T, Oguni H, Yanagaki S, Fukuyama Y, Kogure M, Shimizu H, et al. Chronic localized encephalitis (Rasmussen’s syndrome) preceded by ipsilateral uveitis: a case report. Epilepsia. 1994;35(6):1328–31. 10.1111/j.1528-1157.1994.tb01806.x. PMID: 7988528. 7988528 10.1111/j.1528-1157.1994.tb01806.x

[29] Harvey AS, Andermann F, Hopkins IJ, Kirkham TH, Berkovic SF. Chronic encephalitis (Rasmussen’s syndrome) and ipsilateral uveitis. Ann Neurol. 1992;32(6):826–29. 10.1002/ana.410320621. PMID: 1471877. 1471877 10.1002/ana.410320621

[30] Kashihara K, Ohno M, Takahashi Y. Twenty-one-year course of adult-onset Rasmussen’s encephalitis and bilateral uveitis: case report. J Neurol Sci. 2010;294(1–2):127–30. 10.1016/j.jns.2010.03.016. PMID: 20447655. 20447655 10.1016/j.jns.2010.03.016

[31] Sansevere AJ, Henderson LA, Stredny CM, Prabhu SP, Shah A, Sundel R, et al. Posterior-onset Rasmussen’s encephalitis with ipsilateral cerebellar atrophy and uveitis resistant to rituximab. Epilepsy Behav Rep. 2020;14:100360. 10.1016/j.ebr.2020.100360. 32368732 10.1016/j.ebr.2020.100360 PMC7184158

[32] Kinay D, Bebek N, Vanli E, Gurses C, Gokyigit A, Andermann F. Rasmussen’s encephalitis and Behcet’s disease: autoimmune disorders in first degree relatives. Epileptic Disord. 2008;10(4):319–24. 10.1684/epd.2008.0228. PMID: 19017575. 19017575 10.1684/epd.2008.0228

[33] Korn-Lubetzki I. Rasmussen encephalitis and comorbid autoimmune diseases: a window into disease mechanism? Neurology. 2015;84(16):1721–1721. 10.1212/01.wnl.0000464955.77373.ee. PMID: 25901060. 25901061 10.1212/01.wnl.0000464955.77373.ee

[34] Orsini A, Foiadelli T, Carli N, Costagliola G, Masini B, Bonuccelli A, et al. Rasmussen’s encephalitis: from immune pathogenesis towards targeted-therapy. Seizure. 2020;81:76–83. 10.1016/j.seizure.2020.07.023. PMID: 32769034. 32769034 10.1016/j.seizure.2020.07.023

[35] Cantarín-Extremera V, Jiménez-Legido M, Martín-Rivada Á, Güemes M, Peña-Segura JL, Martínez-González M, et al. Rasmussen’s encephalitis and central precocious puberty. Neuroendocrinological characterization of three cases. Seizure. 2020;83:139–42. 10.1016/j.seizure.2020.10.008. PMID: 33126087. 33126087 10.1016/j.seizure.2020.10.008

[36] Yang Z, Sun G. Headache maybe the initial symptom in Rasmussen’s syndrome: a child case report. Epilepsy Behav Case Rep. 2016;6:75–77. 10.1016/j.ebcr.2016.09.001. 27830116 10.1016/j.ebcr.2016.09.001 PMC5094153

[37] Guo H, Gong P, Yu G, Tang C, Luan G, Liu Q, et al. Three cases of atypical Rasmussen’s encephalitis with delayed-onset seizures. Epilepsia Open. 2025;10(2):411–26. 10.1002/epi4.13136. PMID: 39982333. 39982333 10.1002/epi4.13136 PMC12014926

[38] Caraballo RH, Valenzuela GR, Pociecha J, Princich JP, Gutierrez R, Beltran L, et al. Rasmussen syndrome: an atypical presentation in ten patients. Epileptic Disord. 2018;20(6):468–78. 10.1684/epd.2018.1007. PMID: 30530407. 30530407 10.1684/epd.2018.1007

[39] Gorman KM, Farrell M, Madigan C, King MD, Shahwan A. Rasmussen’s encephalitis, should absence of seizures influence or delay treatment? Childs Nerv Syst. 2015;31(11):2009–10. 10.1007/s00381-015-2917-x. PMID: 26409880. 26409880 10.1007/s00381-015-2917-x

[40] Dupont S, Gales A, Sammey S, Vidailhet M, Lambrecq V. Late-onset Rasmussen encephalitis: a literature appraisal. Autoimmun Rev. 2017;16(8):803–10. 10.1016/j.autrev.2017.05.022. PMID: 28572051. 28572051 10.1016/j.autrev.2017.05.022

[41] Marín-Gracia M, Ciano-Petersen NL, Cabezudo-García P, Fernández-Sánchez V, Salazar-Benítez JA, Muñoz-Zea R, et al. Late-onset Rasmussen encephalitis: 3 illustrative cases and a review of the literature. Neurología (Engl Ed). 2025;40(7):686–99. 10.1016/j.nrleng.2025.07.010. 40903152 10.1016/j.nrleng.2025.07.010

[42] Rizek P, Cheung C, McLachlan RS, Hayman-Abello B, Lee DH, Hammond RR, et al. Childhood-onset nonprogressive chronic encephalitis. Epilepsy Behav. 2014;31:85–90. 10.1016/j.yebeh.2013.11.005. PMID: 24368410. 24368410 10.1016/j.yebeh.2013.11.005

[43] Dandekar S, Wijesuriya H, Geiger T, Hamm D, Mathern GW, Owens GC. Shared HLA class I and II alleles and clonally restricted public and Private brain-infiltrating αβ T cells in a cohort of Rasmussen encephalitis surgery patients. Front Immunol. 2016;7(DEC). 10.3389/fimmu.2016.00608. PMID: 28066418. 10.3389/fimmu.2016.00608 PMC5165278 28066418

[44] Liba Z, Vaskova M, Zamecnik J, Kayserova J, Nohejlova H, Ebel M, et al. An immunotherapy effect analysis in Rasmussen encephalitis. BMC Neurol. 2020;20(1):359. 10.1186/s12883-020-01932-9. PMID: 32972372. 32972372 10.1186/s12883-020-01932-9 PMC7517818

[45] Carreño M, Martí MJ, Aldecoa I, Painous C, Conde E, Valldeoriola F, et al. Unilateral pallidal stimulation for disabling dystonia due to Rasmussen’s disease. J Neurol, Neurosurg Psychiatry. 2019;90(1):108–10. 10.1136/jnnp-2018-318029. PMID: 29986904. 29986904 10.1136/jnnp-2018-318029

[46] Shaikh AT, Das A, Garg A, Radhakrishnan D, Pandit AK, Agarwal AK, et al. Hemichorea in Rasmussen’s encephalitis—a rare case. Mov Disord Clin Pract. 2022;9(4):542–45. 10.1002/mdc3.13439. 35586524 10.1002/mdc3.13439 PMC9092755

[47] Granata T, Gobbi G, Spreafico R, Vigevano F, Capovilla G, Ragona F, et al. Rasmussen’s encephalitis early characteristics allow diagnosis. Neurology. 2003;60(3):422–25. 10.1212/WNL.60.3.422. 12578922 10.1212/wnl.60.3.422

[48] Liu D, Guan Y, Zhou J, Zhai F, Chen L, Li T, et al. The influencing factors and changes of cognitive function within 40 Rasmussen encephalitis patients that received a hemispherectomy. Neurol Res. 2022;44(8):700–07. 10.1080/01616412.2022.2039526. PMID: 35172696. 35172696 10.1080/01616412.2022.2039526

[49] Rudebeck SR, Shavel-Jessop S, Varadkar S, Owen T, Cross JH, Vargha-Khadem F, et al. Pre- and postsurgical cognitive trajectories and quantitative MRI changes in Rasmussen syndrome. Epilepsia. 2018;59(6):1210–19. 10.1111/epi.14192. PMID: 29750339. 29750339 10.1111/epi.14192

[50] Guimarães CA, Souza EAP, Montenegro MA, Marques Jr. JFC, Cendes F, Guerreiro MM. Rasmussen’s encephalitis: the relevance of neuropsychological assessment in patient’s treatment and follow up. Arq. Neuro-Psiquiatr. 2002;60(2B):378–81. 10.1590/s0004-282x2002000300007. PMID: 12131935. 10.1590/s0004-282x2002000300007 12131935

[51] Varadkar S, Tisdall M. Language after Hemispherotomy in Rasmussen syndrome. Pediatr Neurol Briefs. 2016;30(2):13. 10.15844/pedneurbriefs-30-2-4. PMID: 27053909. 27053909 10.15844/pedneurbriefs-30-2-4 PMC4821835

[52] Schulze-Bonhage A, Steinhoff B, Garcés M, Hirsch M, Villanueva V. Efficacy of add-on Cenobamate treatment in refractory epilepsy due to Rasmussen’s encephalitis. Epilepsia Open. 2024;9(6):2537–45. 10.1002/epi4.13060. PMID: 39388362. 39388362 10.1002/epi4.13060 PMC11633691

[53] Lagarde S, Boucraut J, Bartolomei F. Medical treatment of Rasmussen’s encephalitis: a systematic review. Revue Neurologique. 2022;178(7):675–91. 10.1016/j.neurol.2022.01.007. PMID: 35131107. 35131107 10.1016/j.neurol.2022.01.007

[54] Mochol M, Taubøll E, Sveberg L, Tennøe B, Berg Olsen K, Heuser K, et al. Seizure control after late introduction of anakinra in a patient with adult onset Rasmussen’s encephalitis. Epilepsy Behav Rep. 2021, Jan;16:100462. 10.1016/j.ebr.2021.100462. 34189453 10.1016/j.ebr.2021.100462 PMC8219739

[55] Arcan A, Kızılkılıç EK, Gündüz A, Unkun R, Vezzani A, Özkara Ç. Rasmussen’s syndrome treated with anakinra. J Neurol. 2024;271(2):723–26. 10.1007/s00415-023-12072-8. PMID: 37922068. 37922068 10.1007/s00415-023-12072-8

[56] Tekgul H, Polat M, Kitis O, Serdaroglu G, Tosun A, Terlemez S, et al. T-cell subsets and interleukin-6 response in Rasmussen’s encephalitis. Pediatr Neurol. 2005;33(1):39–45. 10.1016/j.pediatrneurol.2005.01.007. PMID: 15876522. 15876522 10.1016/j.pediatrneurol.2005.01.007

[57] Lagarde S, Villeneuve N, Trébuchon A, Kaphan E, Lepine A, McGonigal A, et al. Anti–tumor necrosis factor alpha therapy (adalimumab) in Rasmussen’s encephalitis: an open pilot study. Epilepsia. 2016;57(6):956–66. 10.1111/epi.13387. PMID: 27106864. 27106864 10.1111/epi.13387

[58] Goyal M, Cohen ML, Bangert BA, Robinson S, Singer NG. Rasmussen syndrome and CNS granulomatous disease with NOD2/CARD15 mutations. Neurology. 2007;69(7):640–43. 10.1212/01.wnl.0000267429.89675.03. PMID: 17698784. 17698784 10.1212/01.wnl.0000267429.89675.03

[59] Stredny CM, Steriade C, Papadopoulou MT, Pujar S, Kaliakatsos M, Tomko S, et al. Current practices in the diagnosis and treatment of Rasmussen syndrome: results of an international survey. Seizure Eur J Epilepsy. 2024;122:153–64. 10.1016/j.seizure.2024.09.001. PMID: 39426198. 10.1016/j.seizure.2024.09.001 39426198

[60] Jagtap SA, Patil S, Joshi A, Kurwale N, Jain V, Deshmukh Y. Rituximab in Rasmussen’s encephalitis: a single center experience and review of the literature. Epilepsy Behav Rep. 2022;19:100540. 10.1016/j.ebr.2022.100540. 35509501 10.1016/j.ebr.2022.100540 PMC9058598

[61] Kebir H, Carmant L, Fontaine F, Béland K, Bosoi CM, Sanon NT, et al. Humanized mouse model of Rasmussen’s encephalitis supports the immune-mediated hypothesis. J. Clin. Invest. 2018;128(5):2000–09. 10.1172/JCI97098. PMID: 29629902. 29629902 10.1172/JCI97098 PMC5919802

[62] Browner N, Azher SN, Jankovic J. Botulinum toxin treatment of facial myoclonus in suspected Rasmussen encephalitis. Mov Disord. 2006;21(9):1500–02. 10.1002/mds.20991. PMID: 16758485. 16758485 10.1002/mds.20991

[63] Lozsadi DA, Hart IK, Moore AP. Botulinum toxin a improves involuntary limb movements in Rasmussen syndrome. Neurology. 2004;62(7):1233–34. 10.1212/01.WNL.0000118283.51400.7A. PMID: 15079040. 15079040 10.1212/01.wnl.0000118283.51400.7a

[64] Bingaman JR, Sundar SJ, Hsieh JK, Lu E, Jehi L, Wyllie E, et al. The clinical utility of surgical histopathology in predicting seizure outcomes in patients with Rasmussen encephalitis undergoing hemispherectomy. World Neurosurg. 2022, Jun;162:e517–25. 10.1016/j.wneu.2022.03.043. PMID: 35306199. 35306199 10.1016/j.wneu.2022.03.043 PMC9177654

